# Supporting patients using a digital self-management intervention for symptoms of fatigue, pain, and urgency/incontinence in Inflammatory Bowel Disease: a mixed methods process evaluation of trial facilitators

**DOI:** 10.1371/journal.pone.0350560

**Published:** 2026-06-12

**Authors:** Vari Wileman, Wladyslawa Czuber-Dochan, Rona Moss-Morris, Lesley Dibley, Serena McGuinness, Laura Miller, Christine Norton, Stephanie J.C. Taylor

**Affiliations:** 1 Department of Psychology, School of Mental Health and Psychological Sciences, Institute of Psychiatry, Psychology and Neuroscience, King’s College London UK; 2 Florence Nightingale Faculty of Nursing, Midwifery and Palliative Care, King’s College London UK; 3 Centre for Chronic Illness and Ageing, University of Greenwich, London United Kingdom; 4 Wolfson Institute of Population Health, Queen Mary University of London, United Kingdom; Shahid Beheshti University of Medical Sciences, IRAN, ISLAMIC REPUBLIC OF

## Abstract

**Background:**

A randomised controlled trial (RCT) of a facilitator-supported digital programme (IBD-BOOST) for symptom management in Inflammatory Bowel Disease found no difference in disease-specific quality of life and symptom relief compared with controls. Process evaluation is necessary to inform the interpretation of trial outcomes. This study evaluated facilitator adherence to intervention delivery and facilitator perspectives on training, intervention delivery and supervision during the trial, and implementation.

**Methods:**

A mixed-methods process-evaluation study nested within an RCT. A randomised sample of participant-facilitator in-programme messages during the intervention were assessed using a bespoke fidelity assessment framework. Semi-structured interviews were conducted with nineteen facilitators pre and post intervention delivery and assessed using reflexive thematic analysis.

**Results:**

Facilitators adhered to the trial protocol for intervention delivery achieving high fidelity throughout. Qualitative interviews revealed facilitators valued the IBD-BOOST intervention and felt the training and on-going supervision equipped them well for the task. Facilitators expressed concerns regarding workload-related time constraints during the trial and cautioned that similar implementation challenges may arise if the intervention were delivered within routine clinical care.

**Conclusion:**

This process evaluation demonstrated that, although the overall trial outcomes were unsuccessful, this was not attributable to facilitator infidelity in intervention fidelity or a lack of enthusiasm for the intervention. Instead, other factors, including participant engagement, may have played a significant role in the limited effectiveness observed. Further investigation is needed to identify and address these contributing factors, which could include barriers to participant engagement or potential misalignment between the intervention design and the needs and expectations of its target population.

## Background

Inflammatory Bowel Disease (IBD), including Crohn’s disease and ulcerative colitis, is a complex, long-term condition with chronic inflammation of the gastrointestinal tract; it is associated with multiple symptoms and often co-existing psychological morbidity, including depression and anxiety [1]. Many people with IBD experience pain, fatigue, and faecal urgency/incontinence symptoms; in a recent UK survey of people with IBD (n = 8,486), 42% reported the need for help with their pain, 56% for fatigue and 53% for faecal incontinence; a third of respondents wanted help for all three symptoms [2]. Symptoms often persist even when inflammation is absent or relatively mild and therefore people with IBD might benefit from a more integrated treatment approach, combining clinical treatment with psychosocial support.

**Fig 1:**
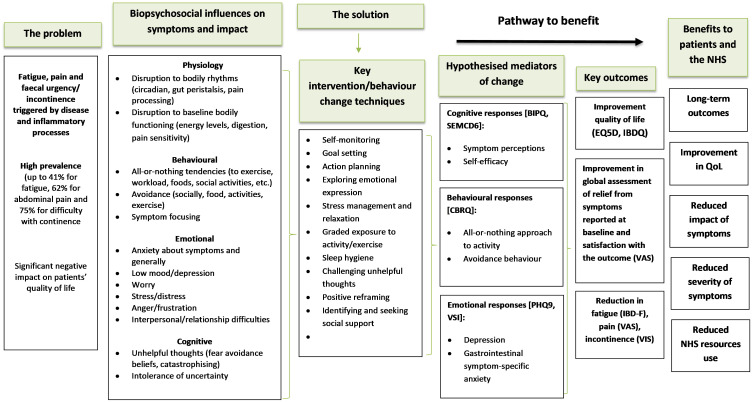
The IBD-BOOST intervention logic model [reproduced under the terms of Creative Commons Attribution 4.0 License 4.0].

The IBD-BOOST intervention is a digital self-management programme (12 sessions) for symptom management, supported by trained facilitators. Based on a cognitive behavioural theoretical framework, the intervention, the first of its kind to address multiple IBD symptoms simultaneously, helps people with IBD make sense of how they experience and cope with symptoms of fatigue, pain, and faecal urgency/incontinence [3]. Cognitive behavioural theory recognises that while disease-related inflammation initiates symptoms, the perception and experience of those symptoms are often shaped by the interaction of cognitive, emotional, and behavioural factors [4]. An intervention development paper describes the intervention theory and logic model in full (Fig 1) [5]. In brief, it was hypothesised that the intervention would enable people with IBD to develop alternative thought processes related to their symptoms and more consistent behaviours, such as balancing activity levels to manage fatigue, which would lead to improved experience of persistent symptoms and overall quality of life. Trained facilitators conducted a 30-minute phone call to guide trial patients’ understanding of cognitive-behavioural factors contributing to their own symptom experience; facilitators then provided weekly in-programme messages to support patients as they progressed through the programme sessions.

The IBD-BOOST intervention was recently evaluated in a large-scale randomised controlled trial (ISRCTN71618461), in which 780 participants were randomised to receive either the IBD-BOOST intervention (n = 391) or usual care (n = 389) [3].At the six-month endpoint, the intervention did not demonstrate significant improvements in the primary outcomes, including IBD-related quality of life and global rating of symptom relief, compared with the control group. However, participants who achieved the predefined minimum intervention dose (≥4 sessions) demonstrated improvements in both IBD-related quality of life and global symptom relief.

Process evaluation can help explain why interventions do or do not achieve their intended outcomes. For example, while the underlying theory of change may be sound, the intervention might not have been delivered or received as planned. Comprehensive fidelity assessments is therefore an important component of intervention evaluation, enabling researchers to assess delivery and receipt, refine intervention content and training, and ultimately optimise implementation. The National Institutes of Health Behaviour Change Consortium framework conceptualises fidelity across five domains: design, training, delivery, receipt, and enactment [6]. This mixed-methods process evaluation focusses on facilitator training, intervention delivery fidelity and facilitators’ experiences of delivering the intervention, including perceived barriers to participant engagement. A separate process evaluation reports on the experiences of trial participants, including intervention receipt and enactment, within the IBD-BOOST trial [7].

The IBD-BOOST intervention was originally designed to be supported by IBD nurse specialists only [5]. However, the COVID-19 pandemic diverted resources to priority patient care reducing the number of nurses available to support the intervention. The trial protocol was adapted to include some facilitators with a psychology background. This adjustment enabled the process evaluation to explore any variations between facilitators with different professional backgrounds which is beneficial for wider research. The potential for IBD nurses to provide psychologically informed support for symptom management has been acknowledged widely [5,8] but IBD nurses have also expressed a ‘lack of confidence’ about their psychological skills and appropriate training, and on-going support is important [9]. Similar concerns are reported across other specialisms addressing long-term conditions [10,11]. Incorporating psychological approaches within clinical practice requires an adjustment to the traditional nursing role of problem-solver, to one which supports patients as experts in their own illness experience, guiding patients towards achieving their goals rather than purely giving advice or instructions. Conversely, facilitators with a psychology background working with clinical populations need an understanding of the clinical aspects of patients’ condition to provide holistic, contextually informed psychological support.

The evidence for digital psychological interventions in IBD is limited, with digital cognitive behavioural therapy studies reporting low engagement and no improvement in quality of life or disease measures [12–14]. However, these studies were self-directed with no facilitator support. Process evaluation studies of facilitators supporting digital psychological interventions for people with IBD and other long-term conditions, report high fidelity to intervention delivery [15]. As scalable digital interventions increasingly support people with long-term conditions, it is essential to evaluate the role of facilitators and the potential added benefits they provide. This study therefore aims to provide evidence from the largest digital intervention in IBD, evaluating whether the facilitator intervention was delivered as intended and exploring facilitators’ views on enablers, barriers, and future implementation.

## Methods

### Study design and setting

This mixed-methods process evaluation was nested within a randomised controlled trial evaluating a self-directed digital programme for people with IBD (n = 780), supported by trial facilitators: the IBD-BOOST trial [Reg: https://doi.org/10.1186/ISRCTN71618461] [16]. A summary of the IBD-BOOST programme is provided in S1 File; further details of the intervention have been reported elsewhere [3,5]. Favourable ethical approval was granted by NRES Research Ethics Committee and the Health Research Authority (London – Surrey Research Ethics Committee/19/LO/0750).

### Participants and sampling

This study employed a total population sampling approach, with data obtained from all facilitators involved in the trial. Facilitators were recruited between 6 January 2020 and 4 February 2022 and included IBD nurse specialists from participating UK National Health Service (NHS) outpatient clinics, as well as a research nurse and psychology graduates recruited from King’s College London. All facilitators provided written informed consent and received training in delivery of the IBD-BOOST intervention. For the fidelity analysis, a random sample comprising 20% of the intervention delivery data was examined, in line with recommended guidelines [17].

### Facilitator training and supervision

Four 1-hour training sessions based on the IBD-BOOST facilitators’ training manual (available on request) were delivered via Microsoft Teams in small groups of 3–4 facilitators (Table 1). Facilitators were trained in the cognitive behavioural approach for IBD symptom management, and the intervention protocol. Competency was assessed using the checklist prompt sheet (S2 File) by facilitator supervisors (LS and VW) via a supervised practice telephone session with a patient volunteer. Facilitators attended monthly group supervision (via Microsoft Teams) with the intervention development team (RMM, VW, LS) to receive support and guidance on supporting patient issues and received one-to-one supervision/support as required.

**Table 1:** IBD-BOOST facilitator remote training and supervision.

Stage of Training	Overview	Details
**Stage 1**	Reading materials	Facilitator training manual provided prior to training.
**Stage 2**	Overview of BOOST RCT Introduction to CBT	1-hour training session: Interactive presentation + exercises.
**Stage 3**	Navigating the BOOST website	1-hour training session: Interactive presentation + exercises.
**Stage 4**	Facilitator-Patient contact & CBT Skills Training (part 1)	1-hour training session: Interactive presentation + exercise. Therapeutic skills and role plays. Homework practice of role play using training materials.
**Stage 5**	Facilitator-Patient contact & CBT Skills Training (part 2)	1-hour training session: In-site messaging. Therapeutic skills and role plays. Homework practice of role play using training materials.
**Practice Patient**	Practice patient exercise Supervision	30-minute audio recorded telephone session and in-site messages with volunteer with IBD. Competencies during phone call assessed by supervisor: guided discovery (prompts and tasks to help patients reflect and discover insights and solutions) ii) empathy and sensitivity iii) validation (acknowledging and accepting someone’s thoughts, feelings, beliefs, and experiences as valid and understandable).
**Supervision**	Group Supervision Individual Supervision	Group supervision (monthly) with supervisor/s to review patient cases and phone call/messaging queries. As required

### Intervention delivery – Facilitator role

Facilitators completed a 30-minute telephone session and guided patients to set meaningful goals for the programme. A checklist prompt sheet was provided to guide the session and enhance fidelity to the intervention (S3 File). Facilitators reviewed their allocated patients’ progress with sessions (with patient written informed consent) accessed via the facilitator platform and supported them with weekly in-programme messages (Table 2) for the duration of the programme (maximum of 12 weeks).

**Table 2:** Protocol for facilitator in-programme messages to patients.

Message protocol
Messages sent when patient does not register
Welcome message sent
Messages sent when patient does not respond
Message sent to arrange telephone session
Message sent to confirm annual leave
Message sent to confirm facilitator access ending
Responds appropriately to medical queries
**Message content based on a cognitive behavioural style**
Summarises content from patient and uses reflection in messages
Expresses encouragement and rewards patient on progress
Demonstrates empathy and sensitivity, and an understanding of the patient’s symptoms and impact
Guiding patient’s understanding of a cognitive behavioural model of symptoms/uses guided discovery techniques in language
Optimises patient engagement and motivation to complete sessions/tasks by highlighting relevant sessions and encouraging progress

*Facilitators omitted messages that were unnecessary given the patient’s progress or if a patient preferred to complete the programme without on-going facilitator support. The content and language style of the messages needed to reflect a cognitive behavioural approach which was a core part of facilitator training and outlined in the manual* (S6 Fig.).

### Data collection

**Fig 2:**
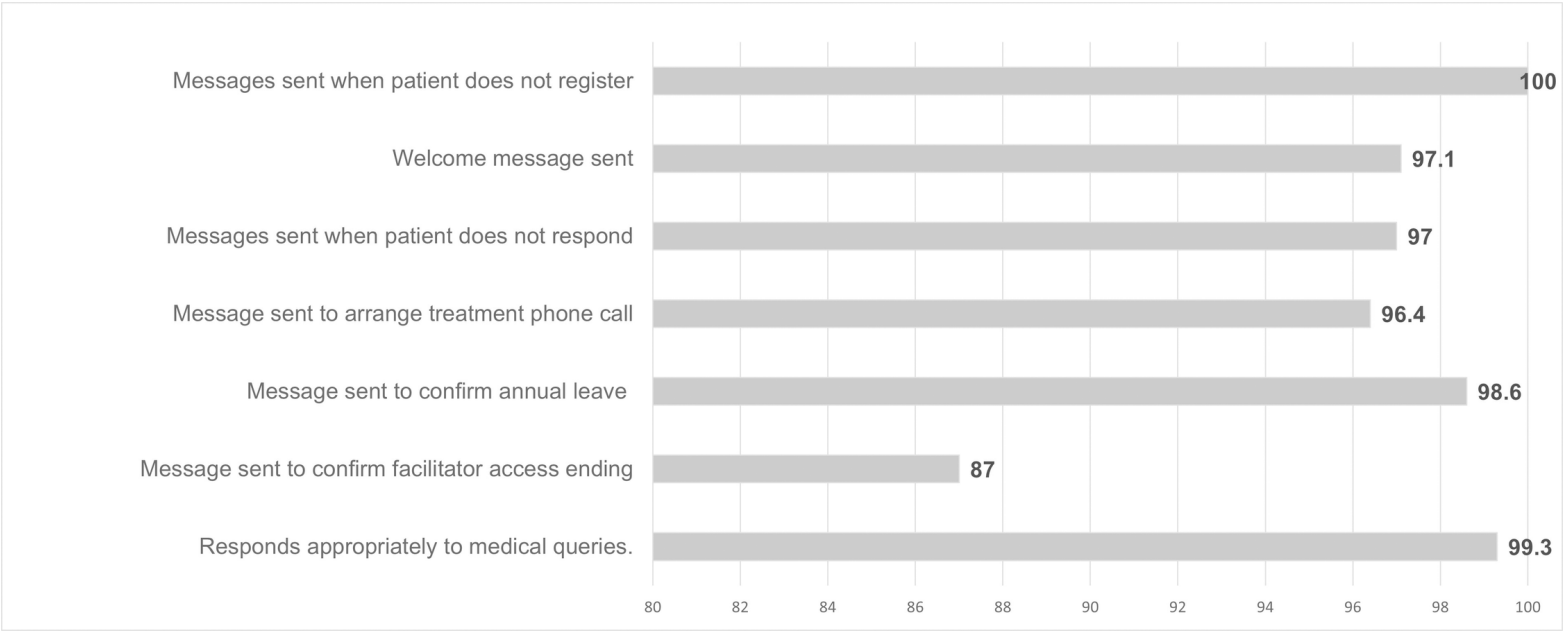
Adherence to the trial protocol for sending messages – percentage of cases achieving fidelity.

### Facilitator intervention delivery and supervision attendance

#### In-programme messages.

A randomised sample of anonymised in-programme messages was assessed for adherence to the trial protocol. Identifiable data (names including facilitator, patient or any other name mentioned in the message, IBD hospital or location) were deleted. Of 391 intervention group patients, messages for the first eighty patients were not retrievable from the database due to an unexpected database error, therefore 311 sets of messages between each patient and facilitator were available. Up to five cases from each facilitator were included in the analyses; where facilitators had a total caseload of five or fewer participants, messages to all participants were included. Where facilitators had caseloads greater than five, a random sample of five patients was selected. This resulted in a final sample of sixty-nine complete sets of messages. Randomisation was conducted anonymously by an independent research team at London North West NHS Trust. The mean number of completed sessions collated by fidelity sample appeared equivalent to the total sample (md = .24, p = .64) and the number of messages sent to and received from their facilitator (md = 1.0, p = .29). Coding was conducted by trial researchers (VW and SM), both trained in psychological interventions and cognitive behavioural methods. Ten cases were randomly selected for duplicate coding by both VW and SM for quality assurance. The remaining cases (*n* = 59) were split between VW (*n* = 30) and SM (*n* = 29) and independently coded.

#### Facilitator interviews.

Facilitators were recruited from participating UK National Health Service (NHS) IBD outpatient clinics (IBD nurse specialists) and from King’s College London (psychology graduates) involved in supporting delivery of the IBD-BOOST intervention. Semi-structured interviews were conducted at two timepoints using predefined topic guides (see S3 File), reflecting the different aims of each interview phase. Pre-intervention interviews were undertaken following facilitator training and explored participants’ professional background, expectations of the facilitator role, perceived preparedness and anticipated challenges in delivering a psychologically informed intervention. Post-intervention interviews explored facilitators’ experiences of intervention delivery, supervision, role development, and perceived barriers and facilitators influencing implementation. Interviews took place between January 2020 and October 2022 and were offered online, in person or via telephone according to participants’ preference (all chose online). Interviews were conducted by one of the two experienced qualitative researchers (WCD and LD), who were independent of both the intervention development and the host randomised controlled trial. Facilitators were invited to talk about their expectations, concerns, and experiences of supporting participants allocated to the intervention arm of the IBD-BOOST trial, including factors that aided or impeded delivery. Topic guides were developed and piloted by the research team prior to data collection to ensure clarity and relevance. All interviews were digitally audio-recorded and transcribed verbatim by an independent professional transcriber. Transcripts were returned to the research team for verification prior to analysis, and all identifiable information (e.g., names of individuals, places or institutions) was removed to ensure confidentiality.

### Data analysis

#### In-programme messages.

A bespoke fidelity framework (S4 File) was developed for evaluating the novel IBD-BOOST intervention structure and assessing the cognitive behavioural approach of in-programme messages to trial patients. A tailored checklist was created using Walton et al.’s five-step process: assessing previous measures (none were appropriate), analysing the intervention, designing a coding system, confirming wording with the team, and piloting [18]. The initial framework was evaluated (using test data not included in the randomised fidelity sample) and refined, in consultation with the trial intervention team. The framework included: 1) Adherence to the trial protocol for sending messages; and 2) Using a cognitive behaviour approach within the message, and a comprehensive checklist of components, which allowed for individual tailoring and expected variability. Components were coded as: 0 (absence of content/inadequate fidelity), 1 (partial fidelity/deviations not appropriate/improvement needed), or 2 (competent fidelity as per protocol including appropriate deviations). Percentage mean (m) and standard deviation (sd) scores were calculated for continuous data. The maximum score for adherence to the trial protocol for sending messages was 14. The maximum score for using a cognitive behavioural approach within the message was 10. Total scores were calculated and reported as a percentage of total adherence. Comparisons between facilitators with a nursing background and facilitators with a psychology background were also conducted using independent samples T-test. Statistical analyses were conducted using STATA statistical software (version 17).

#### Facilitator interviews.

The data were analysed using reflexive thematic analysis to develop pattern of themes from facilitators’ standpoint [19,20]. This approach enabled identification and organisation of both the explicit and latent meanings within the data, supporting the development of meaning-based patterns grounded in facilitators’ experiences of training and intervention delivery rather than researchers’ preconceived assumptions [19,21]. Data analysis followed Braun and Clarke’s six-step approach to reflective thematic analysis recognising the iterative and interpretative nature of the analytic process rather than adherence to a rigid procedural framework [22] (see Table 3). The analysis was undertaken by two researchers (WCD and TYC), independent of both the RCT and the intervention development. Independent coding by two researchers enhanced the credibility and trustworthiness of the study findings [23].Regular discussions with a third researcher (LD) supported refinement and clarification of the themes and sub-themes. NVivo 14 software was used to facilitate data management and analysis.

**Table 3:** Braun and Clarke’s six-step process of reflective thematic analysis and activities undertaken in each step.

Steps of data analysis	Description of the activities
Familiarisation with the data	The data analysis was undertaken by two researchers (WCD and TYC). The researchers familiarised themselves with the data by reading and re-reading the transcripts several times and noting down the initial ideas.
Generating initial codes	Coding the interesting features of the data in a systematic line-by-line fashion across the entire data set, collating data relevant to code with the use of NVivo 14 software for qualitative data analysis. Initially, five pre- and post-intervention interviews were coded independently, then reflected on differences in coding, integrated and constructed the generated coding into a list with conceptualised themes and sub-themes. The list of codes with themes and sub-themes provided a structure that assisted with coding of the remaining transcripts (descriptive analysis).
Searching for themes	Organising codes into potential themes, gathering all data relevant to each potential theme, where the coherent logic order of themes is presenting a ‘story.’ This process was continued inductively coding data with the reference of a pre-developed coding list. Themes and sub-themes were thereby developed by constructing the shared consensuses across transcripts (interpretive analysis). It provided insights into organising and reflecting the core concepts, aiding in creating the logic structure underpinning the compelling interpretation of interview data.
Reviewing themes	Checking if the themes work in relation to the coded extracts (descriptive) and the entire data set (interpretive), generating a thematic ‘map’ of the analysis.
Defining and naming themes	Ongoing analysis to refine the specifics of each theme and the overall story the analysis tells, generating clear definitions and names for each theme.
Producing the report	This step offers a final opportunity for analysis. Selection of vivid, compelling extract examples, final analysis of the selected extracts, relating back to the main aims of the process evaluation, preparing a paper for publication.

### Integration of qualitative and quantitative data

As the study employed a mixed-methods process evaluation design the qualitative and quantitative data relating to facilitator support and intervention delivery were integrated to provide a comprehensive understanding of implementation processes. Quantitative data were used to assess measurable aspects of facilitator training, support and intervention delivery fidelity, while qualitative interview data explored facilitators’ experiences, perceived barriers and contextual influences affecting delivery.

Data integration was undertaken using a triangulation approach, whereby findings from both datasets were compared to identify areas of convergence, complementarity, and divergence [24,25]. Integration primarily occurred at the interpretation stage through narrative weaving, enabling qualitative findings to contextualise and explain quantitative results related to facilitator engagement and implementation outcomes [26]. Consistent with guidance for mixed-methods process evaluations of complex interventions, integrated interpretation of findings is presented within the Discussion section, where comparisons across qualitative and quantitative results are examined to provide explanatory insight into intervention delivery and implementation [27].

## Results

Twenty-six facilitators completed the training; however, ten were unable to proceed with the trial due to COVID-19 redeployment. As a result, sixteen facilitators were able to deliver the intervention to at least one participant. All facilitators were invited to pre- and post- intervention delivery interviews, but some facilitators did not take part in pre- or post-interviews if they commenced intervention delivery before an interview could be arranged or if they were unable to progress with the trial after training and did not deliver the intervention. Nineteen facilitators (three of which did not deliver the intervention) participated in interviews (duration ranged from 17.5 minutes to 86.6 minutes, mean: 53.1 minutes/sd = 15.8). Nine facilitators participated in both pre- and post- intervention interviews. Facilitators consisted of 10 senior nurses (NHS Band 6–8) recruited from UK National Health Service (NHS) IBD outpatient clinics, one research nurse and five psychology graduates – PhD Health Psychology [2], MSc Health Psychology [2], BSc Psychology [1]. Facilitators supported 391 intervention group patients from January 2020 until October 2022; the mean number of patients per facilitator was 24 (sd = 17.9) and ranged from 1–58.

All patients who completed Session 1 were offered the facilitator telephone session; 305 (88%) of 346 patients who completed Session 1 agreed to receive this call. The average call length was 34.3 minutes (sd = 7.5). The mean number of messages sent between patient and facilitator over the 12-week period was 16 (sd = 7.5) of which 10.6 (sd = 4.0) were messages sent from the facilitator to the patient. The mean total hours of facilitators attending group supervision was 11hrs 53 mins (sd = 9hrs 37mins) in addition to the training hours (reported above). Nurse facilitators attended fewer group supervision hours (7hrs 27mins, sd = 7hrs 24mins) than psychology graduate facilitators (21hrs 39mins, sd = 6hrs 4mins) and fewer one-to-one supervision hours (5hrs 36mins, sd = 3hrs, 33mins) than psychology graduate facilitators (11hrs 9mins, sd = 11hrs, 11mins).

### In-programme messages

Facilitators adhered to sending weekly messages through the IBD-BOOST programme with high fidelity in all except five cases (92.8%).

### Adherence to the trial protocol for sending messages

Facilitators achieved fidelity across all components with a mean score was 13.5 (sd = 1.0) out of 14 (Fig 2). Importantly, in cases where patients did not register on the IBD-BOOST programme, there was 100% adherence to the protocol to message patients, encouraging them to register and offer support to commence the intervention programme. The weakest component was “Message sent to confirm facilitator access ending.” The raw data is reported in S5 File.

### Using a cognitive behaviour approach within the message

**Fig 3:**
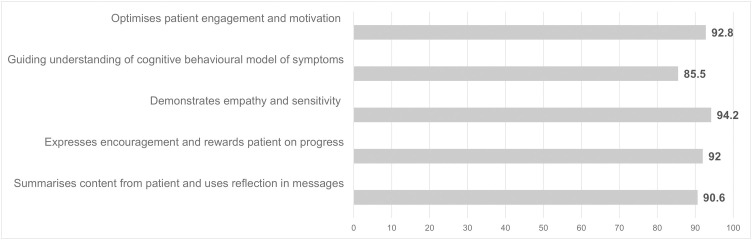
Using a cognitive behaviour approach within the message – percentage of cases reaching fidelity.

Facilitators achieved high fidelity, particularly with demonstrating empathy, sensitivity, and optimising engagement content. The mean total score was 9.2 (sd = 1.6) out of 10 (Fig 3). Facilitators’ ability to use a guided discovery approach to patients was delivered with less competence than the other components.

Nurse facilitators did not significantly differ from psychology graduate facilitators in terms of adherence to the trial protocol for sending messages (md = .43, p = .11) or Using a cognitive behaviour approach within the message (md = .74, p = .06).

### Facilitator interviews

Facilitators strongly supported the IBD-BOOST intervention programme and valued the training and supervision, though concerns about resource constraints during the trial highlighted potential challenges for wider implementation. Nurse facilitators’ motivation stemmed from a belief in the benefits of the self-management approach and a desire to learn CBT skills for everyday practice. However, they also noted difficulties in shifting from their traditional ‘fixer’ mindset to a ‘supporter’ role, emphasising the need for targeted training to address this adjustment.

Three themes and several sub-themes were generated from the data (see Table 4) and are described below supported by verbatim quotes in italics, with facilitator number (F1, F2, etc), their clinical speciality (nurse or psychology graduate) and the interview time point (T1 before intervention or T2 after) in brackets (e.g., F10, Nurse, T1).

**Table 4:** Themes and subthemes identified from pre- and post-intervention facilitator interviews.

Themes	Sub-themes
Challenges, apprehensions, and expectations in the process of becoming facilitator	1.1 Professional background and previous experience (T1) 1.2 Pragmatics of facilitator training (T1) 1.3 Expectations and apprehensions for becoming an IBD-BOOST facilitator (T1)
The learning process led to a clearer understanding of the facilitator role	1.4 The skills and barriers to performing facilitator role (T1 & T2) 1.5 The pragmatics of supervision (T2) 1.6 Practice led to role clarity and confidence (T2)
Recommendations for the intervention and its’ implementation	1.7 The practicality of facilitator role in the intervention (T2) 1.8 Reflections and suggestions on the intervention design (T2) 1.9 Reflections and suggestions on practical issues navigating the online platform (T2) 1.10 Suggestions on supervision (T2) 1.11 Factors that may be considered for future implementation (T2)

Note: T1 and T2 relate to time point one and two of pre- and post-intervention data collection respectively

The themes identified represent a developmental progression in facilitators’ experiences across the intervention delivery process rather than discrete or independent constructs. Theme 1 captures facilitators’ initial positioning as they entered the role, shaped by professional background, prior experience and expectations, alongside apprehensions regarding delivery of a psychologically informed intervention. These early perceptions influenced engagement with training and preparedness for the facilitator role. Theme 2 reflects the transitional learning process through which training, supervision and practical experience supported increasing role clarity, confidence and skill acquisition. This way, facilitators’ understanding evolved from uncertainty to competence as experiential learning occurred during intervention delivery. Building upon this progression, Theme 3 represents a reflective phase, where facilitators drew on their accumulated experience to evaluate the practicality of the role and propose recommendations for intervention refinement and future implementation. Collectively, the themes therefore illustrate an interconnected pathway from initial expectations, through experiential learning, to critical reflection on implementation, demonstrating how facilitator development shaped perceptions of intervention feasibility and sustainability.

### Theme 1: Challenges, apprehensions, and expectations in the process of becoming facilitator

Training was based on CBT principles, intending to help health professionals adapt to the role of ‘facilitator’ in supporting trial participants. This theme captures how facilitators established a fundamental understanding of the role during training. In post training interviews, prior to intervention delivery (T1), facilitators expressed their concerns and expectations for the role.

#### 1.1 Professional background and previous experience.

Facilitators had different clinical backgrounds, with varying skills and knowledge. Some were nurses with IBD experience or research nurses with no previous CBT training, while others were psychology graduates without specific clinical training. Those with nursing experience raised concerns about their ability to adapt to the facilitator role due to a different mindset between being a nurse as a ‘fixer’ and a facilitator role as a ‘supporter.’

*“I think my difficulty will always be just trying not to solve it [the symptoms] for them [patients] because as an IBD nurse I want to fix it because that’s what we do, we are fixers. So that’s going to be my biggest challenge.”* (F7, Nurse, T1)

Facilitators with a nursing background, but without IBD experience, did not perceive this as a barrier to the facilitator role, since their limited IBD knowledge would prevent them from being able to advise on the clinical aspects of care. Facilitators with a psychology background also did not raise lack of IBD knowledge as a concern for adapting to the facilitator role.

*“I haven’t worked with IBD before and my experience that’s relevant to my role here is working in long term conditions and understanding the physical symptom burden and how that relates to the psychological experiences that people often have.”* (F11, Psychology graduate, T1)

#### 1.2 The pragmatics of facilitator training.

Despite some facilitators feeling apprehensive about adapting to the facilitator role due to their clinical background, most expressed positive attitudes towards the overall training and felt well prepared for the role.

*“I found it really reassuring in terms of giving me the feedback that I was going to have the necessary skills to deliver it … the content seemed to pitch perfectly in that it wasn’t too much detail but it stripped back some of the theoretical stuff around CBT and just gave you exactly what you needed to do the role.”* (F11, Psychology graduate, T1)

Most facilitators described how they benefited from the practical aspects of the training, such as role-play and undertaking a practice patient, as well as the support and feedback received from supervisors. They felt that the training helped them to address any mindset conflicts between roles.

*“…like the practice one you do with the patient, I’m quite keen to do that to get the feedback for what I do unconsciously that I think you just need to be a bit mindful throughout. …touching base with supervision throughout will be beneficial*.” (F5, Nurse, T1)

All facilitators were issued with a training manual providing them with the resources to navigate the IBD-BOOST programme and support the patients. Despite the manual being a *‘big document’* that *‘needed time to scour through,’* all facilitators found the training manual very beneficial aiding the training process in skills and knowledge preparation.

“*That’s my ‘go to’ document …all the templates, the guides for the phone call. They give you the beginnings of the sentence and leave you to do the rest and make it very personalised to who you are talking to and what you are talking about.”* (F12, Nurse, T1).

#### 1.3 Expectations and apprehension about becoming a facilitator.

The main motivation for wanting to become a trial facilitator was belief in the self-management approach to symptom management as a method of benefiting patients and wanting to learn CBT skills as an approach to be used in day-to-day practice.

*“I like the idea of the self-care side of it in patients that are clinically in remission but are still having some symptoms that might not always be resolvable … helping patients be realistic about what they can do for themselves and promote that positivity of actually being in control.”* (F8, Nurse, T1)

Facilitators talked about the role expectations and the necessity of having a specific set of therapeutic communication skills and *‘facilitator mindset’* to be able to support patients on their journey through the programme.

*“… helping to motivate patients to continue with the programme, to encourage them to proceed but also to have empathy and understanding with them.”* (F10, Nurse, T1)

*“This is a very different one [role] because we’re not dealing with the causality, we’re helping break the cycle and the management. I found it quite difficult because …the nature of me being a nurse wants me to find the cause of what’s going on.”* (F12, Nurse, T1)

Many facilitators expressed concerns and apprehensions about allocating designated time to the study, in addition to their substantive role and worried about time management and their ability to fulfil the role.

*“When you go into a busy unit it takes one of your colleagues to be off sick that day and that session goes out.”* (F6, Nurse, T1)

### Theme 2: The learning process led to a clearer understanding of the facilitator role

Facilitators talked at length about how they developed a clearer understanding of the role to support the intervention delivery.

#### 2.1 The skills and barriers to performing the facilitator role.

The motivation to become a trial facilitator went beyond their professional time, as many were prepared to dedicate their own personal time (days off and evenings) to fulfil the role, e.g., to attend the training and to run the sessions with the patients. Those who delivered the programme in their own time were remunerated on a sessional basis.

*“I’m doing this outside of my working hours because I feel like there is actually a gap in healthcare service and CBT …I really felt like they [patients] will benefit hugely from CBT model in managing their symptoms.”* (F4, Nurse, T1)

Others tried to incorporate the facilitator’s role into their daily routine or designate a specific time and day each week to create a better structure to support their new additional responsibilities.

#### 2.2 The pragmatics of supervision.

All facilitators had access to individual and group supervision, and this was well received as it provided support, guidance and encouragement. Individual supervision was perceived as *‘immediate’, ‘specific’* and *‘in-depth’* targeting individuals’ needs, feelings and concerns.

*“… quite reflective and constructive and … allow me to gain in confidence, also if you had any anxieties about a patient if they weren’t engaging…it’s nice to talk that through.”* (F6, Nurse, T2)

Group supervision was perceived as an opportunity of *‘learning’*, *‘reflecting’*, and *‘solving’* of broader issues that all facilitators encountered. Many facilitators highlighted the benefits of *‘multidisciplinary’* perspectives and being *‘part of the team’* during the COVID-19 pandemic and working remotely. However, some facilitators found it difficult to share their experience in a group situation, as they felt judged, and for that reason they preferred individual supervision.

*“The individual session feels a bit safer, and you feel less judged. Not that anyone is ever judgemental, but you do feel like if you say something and you’ve got it wrong, I would feel quite uncomfortable.”* (F19, Psychology graduate, T2)

Some facilitators emphasised negative emotions that may arise from the role, and the supervision sessions aided them with support, guidance and the opportunity to reflect.

*“It can be quite difficult supporting people, and I think an important part is to be able to share and say that was a really difficult phone call or a message that was quite distressing or something.”* (F11, Psychology graduate, T2)

#### 2.3 Practice led to role clarity and confidence.

All facilitators mentioned that they became more *‘familiarised’* and *‘confident’* with the role with more practice. Though they experienced a *‘learning curve’* facilitators enhanced their facilitating skills and grew in confidence over time. They felt training was *‘sufficient’* and prepared them well for the role.

*“I think it’s more of a confidence growing exercise for the facilitator and that comes with time.”* (F6, Nurse, T2)

*“It’s sufficient for the purpose, otherwise I think if you knew too much it’s almost like you could possibly step outside the boundaries of what being a facilitator is meant to be, so I think it’s good.”* (F11, Psychology graduate, T2)

Importantly, many facilitators found that initial concerns about conflicts in mindset and skills between facilitator and their speciality background were addressed through practice in intervention delivery.

*“I think I was quite apprehensive about it from the training, but I think the more patients I facilitated, the better that you get.”* (F15, Nurse, T2)

Facilitators grew in confidence and clarity of the role as the study progressed and this was evident in their answers in interviews at T2.

*“Actually, being a facilitator and delivering the study has not been easy, but not as burdensome as I thought. … Helping people to accept it was very enlightening for me as a healthcare professional.”* (F12, Nurse, T2)

### Theme 3: Recommendations for the intervention and its implementation

All facilitators were positive about the self-management intervention with *‘tailored’* (specific to individual’s needs) facilitator support. They also recognised that not all patients needed facilitator support to motivate them to continue with the programme, however, they felt that their support may lead to a *‘higher success rate’*. Facilitators also made several recommendations to improve the intervention and its delivery.

#### 3.1 The practicality of facilitator role in intervention.

All facilitators felt that the telephone session and weekly in-programme messaging had a beneficial effect on patients’ involvement with the programme, helping them to be more motivated and engaged.

*“It’s a crucial part of building the rapport … I think it adds something to the relationship between the patient and the facilitator and then the ongoing messages. I think that’s evidenced by the messages you get back from people that specifically comment on the support that you’ve given them.”* (F11, Psychology graduate, T2)

The telephone session was regarded as *‘crucial’* to provide the overview and clarity of the intervention to the patients, enhancing their engagement and commitment to the programme. Continued support in the form of weekly messaging was seen as valuable to address patient queries and to motivate them.

*“I do think some people probably would be fine just to have the phone call and then just go off and do it themselves, but I don’t know how much they are getting out of it. So yes, I think it’s[messaging] necessary.”* (F19, Psychology graduate, T2)

Some facilitators suggested that after the telephone session, their involvement could be optional instead of necessary as it may not suit everyone, particularly when patients are highly motivated and self-directed. However, others suggested a greater number of telephone sessions (two or three in total) were needed throughout the duration of the intervention to build a stronger relationship and support patients’ engagement.

*“I think some people don’t need a facilitator going through the programme because they’re quite independent. Other people I think like that, and like that accountability, like being prompted to reflect and share further experiences.”* (F19, Psychology graduate, T2).

#### 3.2 Reflections and suggestions on the intervention design.

Facilitators viewed the generic programme sessions (cognitive behavioural content relevant to all symptoms) to be as valuable as the symptom specific sessions on fatigue, pain and urgency/ incontinence.

*“I like the structure. I like the natural progression of the different sessions.… The six core sessions will give you the principles to manage those three, because these are long term symptoms for a patient with IBD. But I also like the fact that they provide symptom specific sessions.”* (F4, Nurse, T2)

Facilitators presented extensive evidence demonstrating that the content of the intervention addressed patients’ needs.

*“I definitely have received a lot of positive messages and it has been amazing really to be part of this journey for a large number of my participants* [patients] *and yes some of whom have had the condition for 20, 30, 40 years and have seen a change, but as I say, I think it’s about just trying to get those people that it’s the right time, it’s the right everything for them.”* (F17, Nurse, T2)

Regarding the digital format of the intervention, all facilitators expressed positive attitudes to the online intervention, as it could be easily *‘accessible’* to many people, providing *‘flexibility’* and *‘convenience’*.

*“I think patients found it good. They could use it at any time of the day. It’s convenient for them, they can do it on the train home.”* (F10, Nurse, T2)

One frequent suggestion was to add extra phone calls mid-programme, and at the end of the programme. Feasibility of adding extra calls were considered by some facilitators in terms of resources and access to patients.

“*It would have been nice to have a final phone call.... It’s not always that easy to arrange the phone call, get access to people’s busy days.”* (F11, Psychology graduate, T2)

A second, frequently suggested change to the programme was to include patient activation measures (e.g., a behavioural assessment tools), that would help to assess the level of patients’ involvement from being passive to being proactive with regards to their symptom self-management. This would help to identify those with different engagement levels and determine if they need facilitator support.

*“We have this in the NHS, as well as in my team, that we assess the patients’ activation measure. So, patients who are really activated they can self-manage, basically they can use the self-management tools. But there are those patients who are also in between, and they need a bit of handholding or a bit of a someone to reassure them.”* (F4, Nurse, T2)

Some facilitators commented on the linear design of the programme potentially contributing to patients losing interest, as the initial more generic sessions may be irrelevant to their needs and not addressing their symptom burden.

*“I think maybe the people that have had a really long diagnosis, they are also the kind of people that would drop off after session two or session three because they’re like oh I know everything. But then they haven’t unfortunately ever then reached sessions that probably would have been most helpful where in the call they’ve maybe said explicitly that I have really irrational thoughts sometimes around my IBD and it stops me from doing things.”* (F19, Psychology graduate, T2)

Hence, to reduce drop-out, the order of the sessions should be less restrictive, giving patients flexibility on which sessions they would like to complete.

#### 3.3 Reflections and suggestions on practical issues navigating the online platform.

Facilitators commented on the practicalities of the facilitator platform; however, their comments were polarised. Some found the platform *‘easy’* to navigate and valued its functions, such as the calendar for booking telephone sessions and checking patients’ engagement in terms of frequency and duration of log-in. Others found the platform *‘clunky’* and suggested that functionality had to be improved.

*“You might be composing a message, and things would time out in the background, and you’d lose the whole message. So, I overcame that by putting messages into Word documents in the first place so that that didn’t happen.” (*F7, Nurse, T2)

#### 3.4 Suggestions on supervision.

A frequently reported barrier in attending the supervision sessions was limited time and capacity. Suggestions were made to add more supervision slots, providing more flexibility for facilitators.

*“I’m not sure if a weekend group supervision might work because obviously it’s quite precious for us working full time but different time slots I think might potentially work.”* (F4, Nurse, T2)

Despite the challenges, facilitators expressed their satisfaction with the online supervision sessions rather than face-to-face, as this provided flexibility and ease of access.

#### 3.5 Factors that may be considered for future implementation.

Facilitators agreed as to the benefit of the intervention, as they perceived it an *‘excellent resource’* for patients, addressing the gap between current healthcare and patients’ unmet needs.

*“I think it [the intervention] takes people through their symptoms step-by-step helping them to understand what’s going on from the physiological and psychological points of view.  ... If done well, if people followed the steps and followed the route then they’d get the maximum benefit from it.”* (F7, Nurse, T2)

Sharing the hopes for the intervention’s beneficial impact on people with IBD affected by fatigue, pain and urgency/incontinence, all facilitators expressed their optimism in the intervention being implemented in the NHS healthcare system.

*“I think it should be part of NHS treatment because mental health has a big impact on patients’ physical wellbeing and vice versa. They are both interactive.”* (F10, Nurse, T2)

Considering the limited NHS resources, some suggested that the intervention could also be implemented via charity routes to enable patients’ access to self-management programme.

*“Some people get these things [symptoms of fatigue, pain, and urgency/incontinence] even when they are clinically in remission. So maybe a separate hub for delivering this facilitator role that separates it from their usual relationship with their IBD nurse might work very nicely.”* (F5, Nurse, T2)

To enable realistic and successful implementation of the intervention, facilitators suggested considering the capacity and structure of the facilitation model and what the facilitators’ background should be.

*“It [supervision model] has to be tailored for that specific IBD service, but because every IBD team is different and it might be useful to open it up, as I said with the right training package and supervision to other members of the healthcare team.”* (F4, Nurse, T2)

## Discussion

This process evaluation study demonstrated that nurses and psychology graduates were sufficiently trained as facilitators of a digital self-management intervention for IBD symptom management and adhered to the protocol delivering the intervention, achieving a high level of fidelity throughout. Qualitative interviews with facilitators found consistent support for the cognitive behavioural intervention approach overall and the IBD-BOOST programme specifically, as it addresses unmet need for many people living with persistent challenging symptoms. The nurse facilitators raised concerns about time restrictions during the trial (due to their competing clinical demands) and suggested that similar challenges could arise if the approach were implemented in NHS IBD clinics. The study findings suggest that although the intervention did not result in significant improvements in primary outcomes, in a randomised controlled trial (RCT), the results were not due to a lack of fidelity in facilitator implementation or a lack of enthusiasm for the programme, and more can be learned from the patient reported feedback on areas to improve including usability and increased facilitator support [7]

We developed a bespoke fidelity evaluation framework that was feasible to conduct within the context of an RCT. The level of facilitator adherence to the trial protocol for in-programme weekly messages was high, with all components achieving considerably higher than 80% fidelity, the minimum expected in a range where 80–100% integrity in intervention delivery typically constitutes high fidelity [17]. Adherence to the trial protocol message instructions was particularly high, with total adherence for messaging trial participants who had not yet registered, a critical task to initiate contact and encourage engagement. The facilitators noted their reliance on the training manual which provided a standardised framework, reducing variability in delivery and enhancing the overall quality and reliability of the intervention, which likely contributed to the high fidelity scores. Conversely, some trial patients did not receive their final message to confirm the end of the facilitator support period. This was unexpected as the trial management team reminded facilitators of this end date and subsequent message but perhaps the importance of this task was not reinforced in training. The successful closure of the facilitator/patient relationship is an important part of the self-management process, reminding the patient of their successes throughout the programme and assuring them of their ability to continue self-management.

Incorporating a cognitive behavioural approach in the message content was also adhered to with high fidelity, although competence in delivering different components varied. Facilitators performed well with *empathy, sensitivity, optimising engagement,* and *encouragement* but less well on *guiding an understanding of the cognitive behavioural model of symptoms*. Guided discovery, an integral cognitive behavioural technique, involves asking open-ended questions and gently challenging assumptions to help patients gain insight into their experiences, in this case their experience of symptoms. Learning and mastering these techniques represents a significant shift from traditional healthcare approaches; facilitating a process where patients arrive at their own conclusions rather than through providing direct advice or instructions. In qualitative interviews, nurse facilitators particularly recognised this need to adapt their usual clinical approach for this purpose. The challenge for health professionals accomplishing guided discovery techniques has been reported previously [28]. However, in a study with respiratory nurses and physiotherapists, there appeared to be an improvement in guided discovery techniques as the trial progressed, suggesting that this is a skill that takes time to develop. Facilitators recognised that increased confidence came with experience and reflected how for some, guided discovery felt like an enlightening experience, stepping away from the role as fixer. Future trials should consider this developmental time and incorporate further training and practice prior to intervention delivery.

Despite some initial apprehension about learning new skills, all facilitators expressed positive attitudes towards the training; they appreciated the *sufficient level* of information, the role play practice, and all felt well prepared for the role. Supervision was seen as invaluable on-going support; the importance of supervision post-training has been reported previously and is particularly valuable for newly trained facilitators learning cognitive behavioural skills [17]. Facilitators noted how individual supervision provided more immediate and in-depth support for specific issues and group supervision provided a supportive team environment where they could share difficult emotions. However, sometimes facilitators felt apprehensive about their contribution in supervision, wanting to *‘get it right.’* Unexpected clinical demands often prevented group supervision attendance which is reflected in the imbalance of supervision time between nurse and psychology graduate facilitators and the amount of supervision should be considered in future trials. Time was a barrier throughout the trial, where many nurse facilitators expressed concerns about finding time to dedicate to the study in addition to their primary clinical responsibilities. This led to worries about time management and the fear that they might not be able to effectively fulfil their role as study facilitators. However, as they were very committed to the success of the study and the potential for the intervention going forward, many were willing to dedicate (paid) personal time to attend training and conduct sessions with patients. Overall, there appears to be a disconnect between the demand for such interventions from both patients and the healthcare professionals who support them, and the concern as to whether such approaches could be implemented in usual care due to resource constraints. Facilitators in the trial reported that more telephone sessions would be appropriate yet in preliminary intervention work, IBD nurses said such a time commitment would not be feasible [5]. Some facilitators suggested that such interventions could be supported by IBD charities, and this could be considered.

Integration of qualitative and quantitative findings provided complementary insight into facilitator support and intervention delivery within the IBD-BOOST trial. Quantitative fidelity data indicated high levels of adherence to the intervention protocol, while qualitative interviews contextualised these findings by highlighting facilitators’ positive engagement with the programme alongside perceived workload pressures and implementation challenges. Together, these datasets suggest that intervention fidelity was maintained despite practical constraints, and that factors beyond facilitator delivery, particularly participant engagement and contextual service pressures, may have influenced trial outcomes. This integrated interpretation strengthens understanding of how intervention processes operated in practice and helps explain the relationship between implementation fidelity and overall intervention effectiveness.

### Strengths and limitations

A key strength of this study is the use of both qualitative and quantitative methods within a mixed-methods process evaluation, enabling comprehensive assessment of facilitator training, support and intervention delivery. The fidelity evaluation followed recommended methodological guidance, involving the development of an intervention content framework, the creation of fidelity checklists and coding guidelines, stakeholder feedback, and piloting and refinement of the assessment procedures [18]. The integration of qualitative and quantitative findings further strengthened interpretation by allowing implementation processes to be examined alongside measurable fidelity outcomes.

Several limitations should be considered when interpreting the findings. Early trial message records were unavailable, preventing assessment of fidelity across the whole sample and potentially introducing bias. Inclusion of these initial cases (n = 80) may have enabled further exploration of the facilitator learning effects over time. However, methodological guidance suggests that early intervention delivery may reasonably be excluded from fidelity sampling while facilitators skills are still developing, meaning this may not represent a substantial limitation [18].The fidelity sample represented 17.6% of the total cases, slightly under the recommended 20% sampling threshold [17]. Fidelity assessment of the telephone sessions was not possible as the calls were not audio-recorded; recording was avoided to minimise potential barriers to participation and preserve openness of discussions. Although facilitators completed checklists confirming call delivery and duration, reliance on self-report represents a methodological limitation that should be addressed in future studies. Facilitators participating in IBD-BOOST, a psychologically informed intervention, may also have been positively predisposed towards the programme. Several facilitators, particularly IBD nurse specialists, expressed interest in developing cognitive behavioural skills for application in routine clinical practice. This may have influenced selection and social desirability bias, potentially contributing to more favourable accounts of intervention delivery. Hence, interviews within the context of an ongoing trial may further have influenced participants’ willingness to express critical perspectives, despite analysis being undertaken by researchers independent of the trial and intervention development. Finally, findings reflect facilitator experiences within UK NHS IBD services and may not be directly transferable to other healthcare settings. Collectively, these limitations may have contributed to a more positive representation of facilitator engagement; however, triangulation with quantitative fidelity data strengthens confidence in the overall interpretation of implementation findings.

### Implications for patient care

Management of long-term conditions remains a significant challenge within contemporary healthcare as people often require psychological as well as medical support. Ongoing debate exists regarding whether such support should be delivered primarily by specialist psychological services or by healthcare professionals trained in psychologically informed approaches within routine clinical care [11]. Whilst both approaches are likely needed, nurses and other healthcare professionals are increasingly being encouraged to adopt psychological approaches in their clinical care. This trend is driven partly by limited access to existing psychological services, characterised by long waiting lists [29,30]. There are also patient-level barriers that make attending these services challenging such as accommodating additional appointments into already demanding treatment schedules, mobility limitations, unexpected illness exacerbations and perceived stigma associated with mental health services [31]. Limited access to specialist psychological services, long waiting times and patient-level barriers, including treatment burden, mobility limitations and perceived stigma, highlight the need for alternative and more accessible models of support.

Findings from this study and other literature suggest that appropriately trained healthcare professionals, particularly specialist nurses, can successfully adopt psychologically informed facilitative roles within digitally supported self-management interventions. [10,11]. However, the NHS has limited resources and healthcare professionals might not have the capacity to fully support patients with all their healthcare needs [5,9,11], so alternative treatment pathways need to be considered, including self-management interventions. With structured training and supervision, both IBD nurses and psychology graduates were able to support intervention delivery with high fidelity, indicating potential for workforce diversification in delivering behavioural interventions. Clear role definition, appropriate supervision and governance structures remain essential, particularly where non-clinically qualified staff are involved.

Our study has found that nurses are willing and competent to undertake psychological roles, furthermore, psychology graduates can support digital health interventions by facilitating user engagement, delivering psychoeducation, and monitoring progress, particularly in settings where nurses or licensed psychologists are unavailable. However, their involvement must be clearly defined, ethically supervised, and limited to non-clinical functions, as they are not qualified to diagnose or provide therapy.

Digitally delivered self-management interventions offer a scalable approach to improving access to psychological and practical support by allowing patients to engage at convenient times and locations. Supported digital interventions may therefore represent an important adjunct to routine care, enabling healthcare professionals to monitor progress, provide encouragement and sustain patient engagement while reducing pressures on specialist services. These findings are likely transferable beyond IBD to other chronic conditions characterised by symptom burden and self-management demands, such as diabetes, chronic pain and rheumatological disorders [32]. Performing a comprehensive evaluation of the intervention delivery in the IBD-BOOST trial and the experiences of facilitators is important for further understanding the trial outcomes but also to contribute to the growing literature evaluating healthcare professionals’ role in delivering psychological approaches in their clinical practice. In our study there were no intervention fidelity differences between nurse and psychology graduate supporting trial participants. Overall, our evaluation suggests that with proper training, guidance, and supervision, both IBD- nurses and psychology graduates can effectively facilitate a digital self-management cognitive-behavioural intervention for IBD-symptom management.

Future research should focus on identifying strategies to optimise patient engagement and intervention uptake, examining sustainable implementation models within routine healthcare systems, and evaluating hybrid workforce approaches combining clinical and non-clinical facilitators. Understanding how digitally supported behavioural interventions can be adapted across long-term conditions will be critical to maximising scalability and long-term clinical impact.

### Conclusion

IBD-BOOST facilitators demonstrated strong commitment to developing skills in delivering a cognitive behavioural self-management approach and valued the training and supervision provided. Despite challenges in transitioning from a traditional ‘fixer’ role to a facilitative ‘supporter’ role, facilitators maintained high fidelity to the programme’s delivery and messaging across all components. These findings indicate that the lack of effectiveness observed in the trial is unlikely to be explained by deficiencies in facilitator engagement, implementation fidelity or a lack of facilitator support for the programme. Rather, the trial results suggest that broader factors, including participant engagement, intervention uptake and contextual service pressures, may have influenced intervention outcomes. This evaluation study makes a novel contribution by providing detailed implementation evidence from a large-scale randomised trial, demonstrating how facilitator training, experiential learning and delivery fidelity operate within digitally supported self-management interventions, thereby advancing understanding of mechanisms underpinning effectiveness and informing optimisation and scalable implementation of complex behavioural interventions in IBD care.

## Supporting information

S1 File The IBD-BOOST programme summary. (PDF)

S2 File Telephone session prompt sheet. (PDF)

S3 File Interview topic guide. (PDF)

S4 File Fidelity coding framework. (PDF)

S5 File Fidelity data detailed results. (PDF)

S6 Fig Example facilitator in-programme patient message. (TIF)

S7 File Raw data file. (PDF)
