# Supplementary material for: Supporting patients using a digital self-management intervention for symptoms of fatigue, pain, and urgency/incontinence in Inflammatory Bowel Disease: a mixed methods process evaluation of trial facilitators
Source: PLoS One. 2026 Jun 12;21(6):e0350560. doi: 10.1371/journal.pone.0350560 (PMC13262822; doi:10.1371/journal.pone.0350560)
Supplement: S1 File — (PDF) [file pone.0350560.s001.pdf]

S1 File. The IBD-BOOST programme summary

Core sessions

**Session 1:** Understanding your IBD symptoms<br>*Task:* symptom diary	• Factors that can contribute to fatigue, pain and urgency in IBD<br>• Identifying factors that relate to you<br>• Use of self-monitoring not symptom focusing<br>• Setting your aims for the programme
**Session 2:**<br>Balancing your activity, eating and exercise<br>*Task:* reviewing goals for activity + sleep diary	• Importance of activity & exercise<br>• How fear leads to avoidance<br>• Eating patterns<br>• Setting your goals for activity and exercise
**Session 3:** Improving your sleep<br>*Task:* reviewing goals for sleep	• Why is sleep important?<br>• Sleep patterns & habits<br>• Improving your sleep<br>• Setting your goals for sleep
**Session 4a:** Changing your thoughts: Part 1<br>*Task:* thought record	• Why are thoughts important?<br>• Identifying unhelpful thinking
**Session 4b:** Changing your thoughts: Part 2<br>*Task: alternative* thought record	• Developing alternative thoughts
**Session 5:** Managing stress and coping with emotions<br>*Task: Reviewing goals for stress + stress diary*	• The effects of stress and finding ways to manage it<br>• The role of emotions and determining how best to take care of yourself<br>• Setting your goals for managing stress and emotions
**Session 6:** Making the most of your social support and communication<br>*Task: Reviewing goals for social support*	• Types of social support<br>• Communication and disclosure<br>• Setting your goals for social support

Symptom-specific sessions

**Session 7:** Managing and understanding fatigue in IBD	• Types of fatigue<br>• Factors related to IBD fatigue<br>• Your vicious cycle of IBD fatigue<br>• Practical strategies to manage fatigue
**Session 8:** Managing and understanding pain in IBD	• What is IBD-pain?<br>• Acute and chronic pain in IBD: what's the difference?<br>• Causes of pain in IBD<br>• A model of IBD pain<br>• How can I best manage my pain?<br>• Common questions around pain in IBD
**Session 9:** Managing and understanding urgency and leakage in IBD	• Bowel functioning and bowel control difficulties<br>• Stress and anxiety in urgency<br>• Exercises to help reduce accidents<br>• Practical bowel management tips<br>• Using social networks to help manage urgency
**Session 10:** The role of acceptance and self-compassion in pain	• What is acceptance and how can it help me?<br>• Role of resilience<br>• Practical exercises

Summary session

**Session 11:** Summary and maintaining improvement	• Reviewing your programme aims<br>• Preparing for the future<br>• Sustaining and building upon improvements
