# Supplementary material for: Supporting patients using a digital self-management intervention for symptoms of fatigue, pain, and urgency/incontinence in Inflammatory Bowel Disease: a mixed methods process evaluation of trial facilitators
Source: PLoS One. 2026 Jun 12;21(6):e0350560. doi: 10.1371/journal.pone.0350560 (PMC13262822; doi:10.1371/journal.pone.0350560)
Supplement: S2 File — (PDF) [file pone.0350560.s002.pdf]

S2 File. The IBD-BOOST telephone session prompt sheet

Session 1 Facilitator Prompt Sheet

Participant Study ID** (first row of your patient details):* | | | | ***Duration of call (mins): | |

Please complete the checklist below after each phone call and scan + email/photocopy + post back to the BOOST team. Thank you.

**Task Type** (time guide)	**Task**	**Completion**
**Introduction to session** (2-3 mins)	Introductions	☐
	Check practicalities and technical issues e.g. vicious cycle	☐
	Set an agenda	☐
**Brief assessment** (5 mins)	Symptoms	☐
	Severity and impact of symptoms on daily activities	☐
	Triggers	☐
**CBT model** (10-15 mins)	Ask about the symptom and what they do in response (or what do they not do)	☐
	Going through personal vicious cycle (thoughts, emotions and behaviours)	☐
	Relate psychological processes to biological processes	☐
	Explain how the programme works (core and symptom-specific sessions)	☐
**Programme aims** (5 mins)	Programme aims in relation to the content	☐
**Closing the session** (2-3 mins)	Recommend weekly log in and check any barriers to intervention (and how you can overcome these)	☐
	Clarify further communication (in-site messaging)	☐
	Additional questions and goodbyes	☐

Facilitator name: **Signed:...Date:....................**
