# Supplementary material for: Supporting patients using a digital self-management intervention for symptoms of fatigue, pain, and urgency/incontinence in Inflammatory Bowel Disease: a mixed methods process evaluation of trial facilitators
Source: PLoS One. 2026 Jun 12;21(6):e0350560. doi: 10.1371/journal.pone.0350560 (PMC13262822; doi:10.1371/journal.pone.0350560)
Supplement: S3 File — (PDF) [file pone.0350560.s003.pdf]

S3 File. The IBD-BOOST telephone session prompt sheet

**Opening question:**	**Thank you for taking part as a facilitator for the intervention. Can you tell me about your expectations or anticipations for this role?**
Listen for and follow-up on any of these aspects:	• *How did you find the training for the facilitator's role?*<br>• *Is there anything else that you would have liked as part of the preparation?*<br>• *What do you think about the overall training in relation to your knowledge of CBT and psychological therapy? Was the training sufficient for you to support the intervention?*<br>• *Will you use the training manual? What do you think of the manual?*<br>• *What do you think you will do well in with this role? What do you think you may not do well in or may need extra support for?*<br>• *How do you feel about the supervision that will be provided during your time as a facilitator?*
**Probing questions:**	**Do you think it will be possible to implement this service into your current work?**
Listen for and follow-up on any of these aspects:	• *How do you think it will work in terms of time allocation? Do you think you will allocate a specific day/time for the trial facilitation, or will this be incorporated into your other activities? What do you think will work best? Do you think you will have any unplanned contacts with the patients taking part in the intervention? Ask about frequency, etc…*<br>• *What comments do you think patients will have in relation to the intervention?*<br>• *Do you think the patients will talk about symptom change (deterioration or improvement) during the intervention period?*<br>• *Do you think you will like to use this intervention with other patients?*<br>• *Do you think you would recommend this intervention to other patients and health professionals to use for symptom management?*
**Probing question:**	**What do you think about the facilitator's role?**
Listen for and follow-up on any of these aspects:	• *Do you think the facilitator's role is required?*<br>• *What would you think if the intervention was run with and/or without the facilitator?*<br>• *Out of the care team members, who do you think are the right people to support the intervention? (Are nurses or other professional groups best suited to support the intervention and why?)*<br>• *How difficult /easy do you think it will be to keep the focus on these three symptoms?*
**Probing question:**	**What is your overall opinion about the intervention?**
Listen for and follow-up on any of these aspects:	• *Do you think the intervention will be helpful in terms of managing patients' symptoms?*<br>• *Do you think the format of the intervention will be appropriate for patients' needs? What do you think about the phone call vs. messaging? Do you think one 30-minute phone call will be sufficient? How much time do you think it will take for you for to do the messaging? What would be your preference? What do you think patients will prefer?*

	• *Do you think the intervention will meet the patients' expectations about managing their symptoms of fatigue, abdominal pain, faecal urgency/incontinence?*<br>• *Do you think the intervention will improve their life? If yes, in what way? If no, why do you think this may be?*<br>• *Would recommend making any changes to the intervention? If yes, what changes would you recommend?*
**Closing question:**	**Is there anything else that you would like to add?**

Post-Intervention Facilitator Interviews

**Opening question:**	**Thank you for facilitating the intervention. Can you tell me about your experience of being a facilitator?**
Listen for and follow-up on any of these aspects:	• *What did you think of the training for the facilitator's role?*<br>• *Is there anything else that you would have liked as part of the preparation?*<br>• *What do you think about the overall training in relation to your knowledge of CBT and psychological therapy? Was the training sufficient for you to support the intervention?*<br>• *How did supervision work throughout the trial? Did you have an individual or group supervision? What were your experiences and perceptions of both? Any barriers to the engagement? What was your understanding of the facilitator's supervision? What role did it have for you?*<br>• *Did you use the training manual? What do you think of the manual?*<br>• *What worked well and what didn't work as expected?*<br>• *What could be improved in terms of the content of the intervention?*<br>• *What do you think about the functionality of the intervention? What do you think about the facilitator's platform in the BOOST programme? What do you think of its functionality? Was it practical? (for the facilitator and the patient?)*
**Probing questions:**	**Do you think it is possible to implement this service into your current work?**
Listen for and follow-up on any of these aspects:	• *How did it work in terms of time allocation? Did you allocate a specific day/time for the trial facilitation, or was this incorporated into your other activities? What do you think would work best? Did you have any unplanned contacts with the patients taking part in the intervention? Ask about frequency, etc… (allocating your own time to do the facilitator's role)*<br>• *What comments did patients have in relation to the intervention?*<br>• *Did the patients talk about symptom change (deterioration or improvement) during the intervention period?*<br>• *Would you like to use this intervention with other patients?*<br>• *Would you recommend this intervention to other patients and health professionals to use for symptom management?*
**Probing question:**	**What do you think about the facilitator's role?**

Listen for and follow-up on any of these aspects:	• *Do you think facilitator's role is required?*<br>• *What would you think if the intervention was run with and/or without the facilitator?*<br>• *Out of the care team members, who do you think are the right people to support the intervention? (Are nurses or other professional groups best suited to support the intervention and why?) Do you think that people with IBD (non-professionals) could be trained in facilitating BOOST?*<br>• *What do you think about peer supervision between the facilitators?*<br>• *How difficult /easy was it to keep the focus on these three symptoms?*
**Probing question:**	**What is your overall opinion about the intervention?**
Listen for and follow-up on any of these aspects:	• *Was the intervention helpful in terms of managing patients' symptoms?*<br>• *Was the format of the intervention appropriate for patients' needs? What do you think about the phone call vs. messaging? Was one 30-minute phone call sufficient? How much time did it take you for the messaging? What would be your preference? What do you think patients preferred?*<br>• *Did the intervention meet the patients' expectations about managing their symptoms of fatigue, abdominal pain, faecal urgency/incontinence? What are your thoughts on the IBD BOOST intervention in relation to helping people with fatigue, pain, faecal urgency/incontinence? Do you think that some people with some symptoms may have benefited more than those with other symptoms?*<br>• *Did the intervention improve their life? If yes, in what way? If no, why do you think this is?*<br>• *Would you recommend making any changes to the intervention? If yes, what changes would you recommend?*
**Closing question:**	**Is there anything else that you would like to add?**
