# Supplementary material for: Supporting patients using a digital self-management intervention for symptoms of fatigue, pain, and urgency/incontinence in Inflammatory Bowel Disease: a mixed methods process evaluation of trial facilitators
Source: PLoS One. 2026 Jun 12;21(6):e0350560. doi: 10.1371/journal.pone.0350560 (PMC13262822; doi:10.1371/journal.pone.0350560)
Supplement: S4 File — (PDF) [file pone.0350560.s004.pdf]

S4. Fidelity coding framework

Coding framework – Facilitator message content<br>Key content and procedures expected to be included/excluded in trial facilitators.<br><br>Message frequency will be calculated as **0** (absence of feature/inadequate fidelity) and **1** (competent fidelity as per protocol).<br><br>Scores will be calculated on as: **0** (absence of feature/inadequate fidelity), **1** (partial fidelity/deviations/improvement needed), or **2** (competent fidelity as per protocol).		
Messages sent weekly as appropriate (Message frequency)	Respond within c.7 working days if you receive an in-site message from a patient (Training manual Page 10). Check if messages are typically sent weekly, mindful of deviations e.g., facilitator annual leave. Check number of messages compared with number of sessions and assess in relation to patient engagement e.g., up to 12 messages for patient who engages for the whole 12 weeks compared with e.g., six weeks of 'checking-in' messages for non-engagement etc (see below) and so on.	0=message frequency inadequate<br>1=message frequency as per protocol
Messages sent when patient does not register	Facilitator sends Welcome message and one further checking-in message one week later. If patient does not register this would then be referred to the trial team for follow-up. Check if two messages are sent for patients who do not register or have not registered within 14 days of allocation.	0= protocol missing or inappropriate<br>1= protocol followed with deviations e.g., second message missing<br>2= protocol followed completely
Welcome message sent	Check message compared with sample protocol message in training manual e.g., description of process/30min phone call after Session 1 completed. Patients sometimes register and complete Session 1 before the facilitator is aware. Assess each case and score on both welcome messages combined.	0= protocol missing or inappropriate<br>1= protocol followed with deviations<br>2= protocol followed completely
Messages sent when patient does not respond	If no response from patient, facilitator sends up to six messages, one per week. After six weeks, facilitator messages patient letting them know that they will pause the messages until closing message.	0= protocol missing<br>1= protocol followed with deviations<br>2= protocol followed completely

Message sent to arrange treatment phone call	Check message compared with sample protocol message in training manual e.g., checking date/time, offering change if not convenient, reminder about vicious cycle, quiet space, confidentiality etc. Do we need to include/consider DNAs, follow-up messages etc? Consider when assessing?	0= protocol missing or inappropriate<br>1= protocol followed with deviations<br>2= protocol followed completely
Message sent to confirm annual leave	Facilitator sends a message to patients explaining absence and that they will not receive messages during this time	0= protocol missing or inappropriate<br>1= protocol followed with deviations<br>2= protocol followed completely
Message sent to confirm facilitator access ending	Facilitator sends a message explaining that the messaging will cease and confirms that the patient still has access to BOOST for a further 3months. Style per template appropriate given patient engagement	0= final closing message missing (check if sent by NHS email on Intervention log).<br>1= protocol followed with deviations e.g., explains facilitator access ending but excludes other content e.g., further 12 weeks and encouragement to review sessions etc<br>2= protocol followed completely
Responds appropriately to medical queries.	Facilitator responds by acknowledging symptoms/medical issue and responds appropriately – recommends IBD medical team, refers other source (e.g., Crohn's & Colitis support)	0= protocol missing or inappropriate e.g., provides extensive medical advice<br>1= protocol followed with deviations e.g., brief reference to medical advice<br>2= protocol followed completely
Applies a cognitive behavioural approach		
***Important context – in BOOST the facilitators are not CBT therapists**	**Summarising/reflecting** – e.g. *"I'm really pleased that you're feeling better this week and that you found the sleep diary helpful"* or *"It sounds like it's been a tough week for you, I'm sorry to hear that…"*, or *"So, you've noticed that your fatigue gets worse when you try and get through lots of things in a short period of time?"*	0= cognitive behavioural approach as outlined in BOOST protocol and training, missing from messages.<br><br>1= some messages reflect a cognitive behavioural approach as outlined in BOOST protocol and training - with some inconsistencies/improvement required.
	**Expresses encouragement and rewards patient on progress** – e.g. "*You've made great progress with the BOOST programme…"* and/or *"Well done for the effort you've put into the BOOST programme; I can see the time you've invested in this.…"*	

	**Demonstrates empathy and sensitivity, and an understanding of the patient's symptoms and impact** e.g., "*Thank you for sharing with me about your current circumstances. I am really sorry to hear you're having a bit of a flare at the moment and that work is particularly stressful, that sounds tough*". Or "*You did so well to visit the café despite feeling anxious beforehand. That can't have been easy, and it sounds like you felt quite relieved that it was a good experience, is that right?*	2= most/all messages reflect a cognitive behavioural approach as outlined in BOOST protocol and training.
	**Guiding patients' understanding of a cognitive behavioural model of symptoms / uses guided discovery techniques in language** (explores ideas, active curiosity, questions, makes links, adopts a collaborative approach). E.g., *"In terms of the thought that you noted about being 'anxious before the conversation develops because it tends to go wrong', can you think of any facts that contradict this or any instances where this wasn't the case?"*	
	**Optimises patient engagement** and motivation to complete sessions/tasks by **highlighting relevant sessions** and **encouraging progress e.g.** *It's understandable that reflecting on difficult thoughts, emotions and past experiences can be tough – well done for persevering with these and I'm so pleased that you are finding some benefit already… you might find the session on acceptance and self-compassion helpful here as it provides some helpful tips on how to be kind to yourself, including keeping a diary…"*	

Coding framework – Facilitator message content

Key content and procedures expected to be included/excluded in trial facilitators.

Message frequency will be calculated as **0** (absence of feature/inadequate fidelity) and **1** (competent fidelity as per protocol).

Scores will be calculated on as: **0** (absence of feature/inadequate fidelity), **1** (partial fidelity/deviations/improvement needed), or **2** (competent fidelity as per protocol).

Messages sent weekly as appropriate (Message frequency)	Respond within c.7 working days if you receive an in-site message from a patient (Training manual Page 10). Check if messages are typically sent weekly, mindful of deviations e.g., facilitator annual leave. Check number of messages compared with number of sessions and assess in relation to patient engagement e.g., up to 12 messages for patient who engages for the whole 12 weeks compared with e.g., six weeks of 'checking-in' messages for non-engagement etc (see below) and so on.	0=message frequency inadequate<br>1=message frequency as per protocol
Messages sent when patient does not register	Facilitator sends Welcome message and one further checking-in message one week later. If patient does not register this would then be referred to the trial team for follow-up. Check if two messages are sent for patients who do not register or have not registered within 14 days of allocation.	0= protocol missing or inappropriate<br>1= protocol followed with deviations e.g., second message missing<br>2= protocol followed completely
Welcome message sent	Check message compared with sample protocol message in training manual e.g., description of process/30min phone call after Session 1 completed. Patients sometimes register and complete Session 1 before the facilitator is aware. Assess each case and score on both welcome messages combined.	0= protocol missing or inappropriate<br>1= protocol followed with deviations<br>2= protocol followed completely
Messages sent when patient does not respond	If no response from patient, facilitator sends up to six messages, one per week. After six weeks, facilitator messages patient letting them know that they will pause the messages until closing message.	0= protocol missing<br>1= protocol followed with deviations<br>2= protocol followed completely
Message sent to arrange treatment phone call	Check message compared with sample protocol message in training manual e.g., checking date/time, offering change if not convenient, reminder about vicious cycle, quiet space, confidentiality etc. Do we need to include/consider DNAs, follow-up messages etc? Consider when assessing?	0= protocol missing or inappropriate<br>1= protocol followed with deviations<br>2= protocol followed completely

Message sent to confirm annual leave	Facilitator sends a message to patients explaining absence and that they will not receive messages during this time	0= protocol missing or inappropriate<br>1= protocol followed with deviations<br>2= protocol followed completely
Message sent to confirm facilitator access ending	Facilitator sends a message explaining that the messaging will cease and confirms that the patient still has access to BOOST for a further 3months. Style per template appropriate given patient engagement	0= final closing message missing (check if sent by NHS email on Intervention log).<br>1= protocol followed with deviations e.g., explains facilitator access ending but excludes other content e.g., further 12 weeks and encouragement to review sessions etc<br>2= protocol followed completely
Responds appropriately to medical queries.	Facilitator responds by acknowledging symptoms/medical issue and responds appropriately – recommends IBD medical team, refers other source (e.g., Crohn's & Colitis support)	0= protocol missing or inappropriate e.g., provides extensive medical advice<br>1= protocol followed with deviations e.g., brief reference to medical advice<br>2= protocol followed completely
Applies a cognitive behavioural approach		
***Important context – in BOOST the facilitators are not CBT therapists**	**Summarising/reflecting** – e.g. *"I'm really pleased that you're feeling better this week and that you found the sleep diary helpful"* or *"It sounds like it's been a tough week for you, I'm sorry to hear that…"*, or *"So, you've noticed that your fatigue gets worse when you try and get through lots of things in a short period of time?"*	0= cognitive behavioural approach as outlined in BOOST protocol and training, missing from messages.<br><br>1= some messages reflect a cognitive behavioural approach as outlined in BOOST protocol and training - with some inconsistencies/improvement required.<br><br>2= most/all messages reflect a cognitive behavioural approach as outlined in BOOST protocol and training.
	**Expresses encouragement and rewards patient on progress** – e.g. "*You've made great progress with the BOOST programme…*" and/or *"Well done for the effort you've put into the BOOST programme; I can see the time you've invested in this.…"*	
	**Demonstrates empathy and sensitivity, and an understanding of the patient's symptoms and impact** e.g., "*Thank you for sharing with me about your current circumstances. I am really sorry to hear you're having a bit of a flare at the moment and that work is particularly stressful, that sounds tough*". Or "*You did so well to visit the café despite feeling anxious beforehand. That can't have been easy, and it sounds like you felt quite relieved that it was a good experience, is that right?*	

	**Guiding patients' understanding of a cognitive behavioural model of symptoms / uses guided discovery techniques in language** (explores ideas, active curiosity, questions, makes links, adopts a collaborative approach). E.g., *"In terms of the thought that you noted about being 'anxious before the conversation develops because it tends to go wrong', can you think of any facts that contradict this or any instances where this wasn't the case?"*	
	**Optimises patient engagement** and motivation to complete sessions/tasks by **highlighting relevant sessions** and **encouraging progress e.g.** *It's understandable that reflecting on difficult thoughts, emotions and past experiences can be tough – well done for persevering with these and I'm so pleased that you are finding some benefit already… you might find the session on acceptance and self-compassion helpful here as it provides some helpful tips on how to be kind to yourself, including keeping a diary…"*	
