# Supplementary material for: Supporting patients using a digital self-management intervention for symptoms of fatigue, pain, and urgency/incontinence in Inflammatory Bowel Disease: a mixed methods process evaluation of trial facilitators
Source: PLoS One. 2026 Jun 12;21(6):e0350560. doi: 10.1371/journal.pone.0350560 (PMC13262822; doi:10.1371/journal.pone.0350560)
Supplement: S5 File — (PDF) [file pone.0350560.s005.pdf]

S5 File. Fidelity data detailed results

Findings: *core content*

Component	Occurrence	Delivery as per protocol	Fidelity given patient context (%)
Messages sent when patient does not register	2	All cases delivered per protocol	100
Welcome message sent	69	3 cases not delivered fully per protocol<br>(1 case scored=0, 2 cases scored =1)	97.1
Messages sent when patient does not respond	22	4 cases not delivered fully per protocol<br>(4 cases scored =1)	97
Message sent to arrange treatment phone call	56	4 cases not delivered fully per protocol<br>(1 case scored=0, 3 cases scored =1)	96.4
Message sent to confirm annual leave	35	1 case not delivered per protocol<br>(1 case scored =0)	98.6
Message sent to confirm facilitator access ending	60	13 cases not delivered fully per protocol<br>(5 cases scored=0, 8 cases scored =1)	87
Responds appropriately to medical queries.	21	1 case not delivered per protocol<br>(1 case scored=0)	99.3

Findings: *applies a cognitive behavioural approach*

Message content	Occurrence (%)	Fidelity given patient context (%)
Summarises content from patient and uses reflection in messages	48 (70)	90.6
Expresses encouragement and rewards patient on progress	67 (97)	92.0
Demonstrates empathy and sensitivity, and an understanding of the patient's symptoms and impact	64 (93)	94.2
Guiding patients' understanding of a cognitive behavioural model of symptoms / uses guided discovery techniques in language	31 (45)	85.5
Optimises patient engagement and motivation to complete sessions/tasks by highlighting relevant sessions and encouraging progress	67 (97)	92.8
