# Supplementary figures and images for: Supporting patients using a digital self-management intervention for symptoms of fatigue, pain, and urgency/incontinence in Inflammatory Bowel Disease: a mixed methods process evaluation of trial facilitators
Source: PLoS One. 2026 Jun 12;21(6):e0350560. doi: 10.1371/journal.pone.0350560 (PMC13262822; doi:10.1371/journal.pone.0350560)

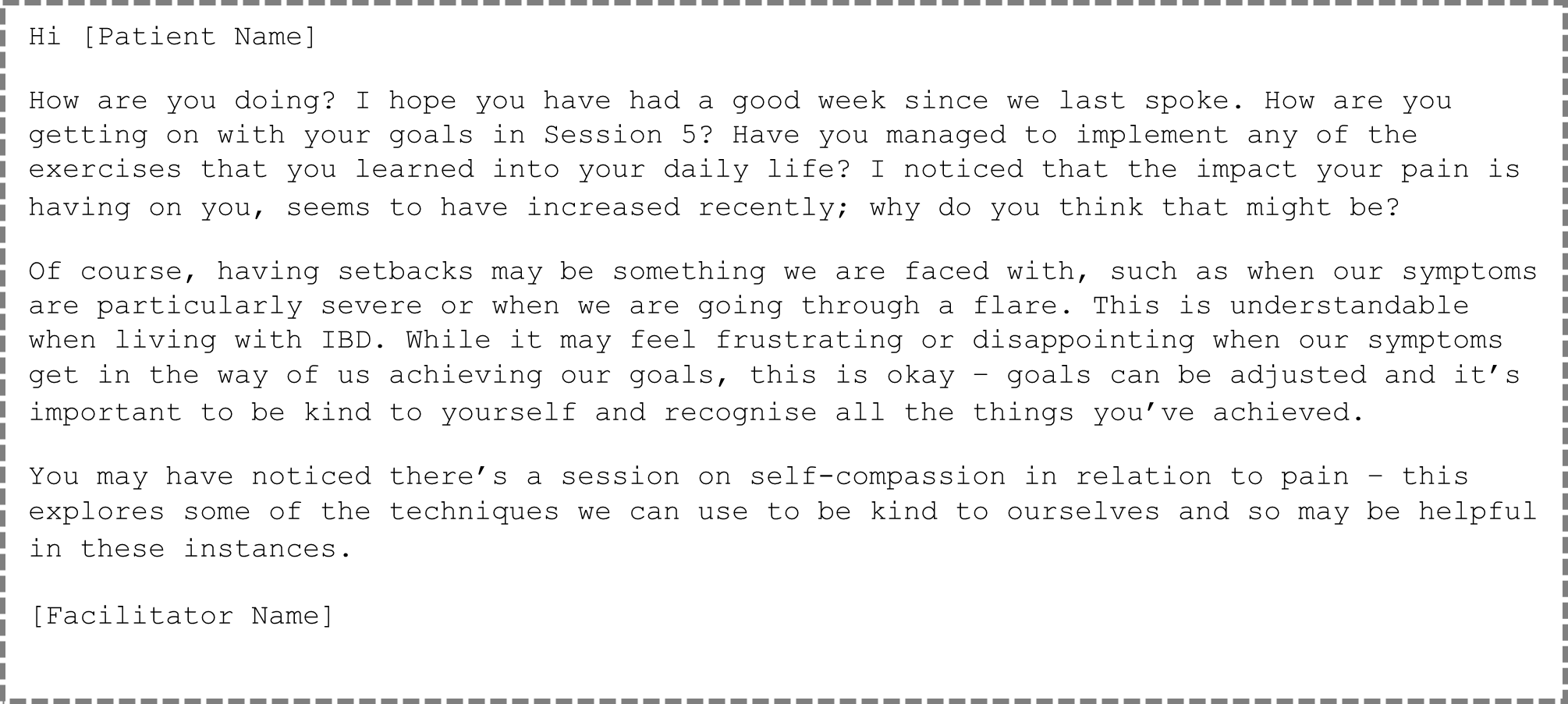

Supplement: S6 Fig — (TIF) [file pone.0350560.s006.tif]
