# Supplementary material for: Supporting patients using a digital self-management intervention for symptoms of fatigue, pain, and urgency/incontinence in Inflammatory Bowel Disease: a mixed methods process evaluation of trial facilitators
Source: PLoS One. 2026 Jun 12;21(6):e0350560. doi: 10.1371/journal.pone.0350560 (PMC13262822; doi:10.1371/journal.pone.0350560)
Supplement: S7 File — (PDF) [file pone.0350560.s007.pdf]

S7 File. Raw data file

CASE NUMBER	CASE ID	Patient not registering Did/did not happen 0=no 1=yes	Patient not registering Reason did not happen	Patient not registering (0,1,2)	Welcome msg Did/did not happen 0=no 1=yes	Welcome msg Reason did not happen	Welcome msg (0,1,2)	Patient not responding Did/did not happen 0=no 1=yes	Patient not responding Reason did not happen	Patient not responding (0,1,2)	Arranging treatment call Did/did not happen 0=no 1=yes	Arranging treatment call Reason did not happen	Arranging treatment call (0,1,2)	Annual leave msg Did/did not happen 0=no 1=yes	Annual leave msg Reason did not happen	Annual leave msg (0,1,2)	Ending message Did/did not happen 0=no 1=yes	Ending message - Reason did not happen	Ending message (0,1,2)	Response to medical content Did/did not happen 0=no 1=yes	Response to medical content Reason did not happen	Response to medical content (0,1,2)	Summarising/ reflecting Did/did not happen 0=no 1=yes	Summarising/ reflecting Reason did not happen	Summarising/ reflecting (0,1,2)	Encourages and rewards Did/did not happen 0=no 1=yes	Encourages and rewards Reason did not happen	Encourages and rewards (0,1,2)	Empathy/ understanding Did/did not happen 0=no 1=yes	Empathy/ understanding Reason did not happen	Empathy/ understanding (0,1,2)	Guided discovery_ CBA model Did/did not happen 0=no 1=yes
Count		2		138	69		134	22		134	56		133	35		136	60		120	21		137	48		125	67		127	64		130	31
Percentage of cases				100			97.1			97.1			96.4			98.6			87.0			99.3	69.6		90.6	97.1		92.0	92.8		94.2	44.9
1	118	0		2	1		2	1	Minimal pa	2	1		2	1		2	1		2	0		2	1		2	1		2	1		2	0
2	136	0		2	1		2	0		2	1		2	1		2	1		2	0		2	1		2	1		2	1		2	1
3	139	0		2	1		2	0	Minimal pa	2	0	Minimal pa	2	0		2	1		2	0		2	1	Minimal pa	1	1		2	1		2	0
4	140	0		2	1		2	0		2	1		2	0		2	1		2	1		2	1		2	1		2	1		2	1
5	144	0		2	1		2	0		2	1		2	1		2	1		2	0		2	1		2	1		2	1		2	1
6	155	0		2	1		2	0		2	1		2	0		2	1		1	1		2	1		1	1		1	1		1	1
7	156	0		2	1		2	0		2	1		2	1		2	0	Patient wi	2	0		2	1		2	1		2	0	Patient wi	2	0
8	194	0		2	1		2	0		2	1		2	0		2	1		2	1		2	1		2	1		2	1		2	1
9	199	0		2	1		2	1		2	1		1	1		2	1		1	0		2	0	Minimal pa	1	0		0	1		0	0
10	203	0		2	1		2	0	Minimal pa	2	1		2	1		2	1		2	0		2	1	Minimal pa	2	1		2	1		2	0
11	209	0		2	1		2	0		2	1		2	1		2	1		2	0		2	1		2	1		2	1		2	1
12	212	0		2	1		2	0	Minimal pa	2	1		2	1		2	1		2	0		2	1		2	1		2	1		1	0
13	218	0		2	1		2	0		1	1		1	1		2	0		0	0		2	0	Minimal pa	1	1		1	1		2	0
14	223	0		2	1		2	0		2	1		2	1		2	1		2	1		2	1		2	1		2	1		2	1
15	224	0		2	1		2	0		2	1		2	0		2	1		2	0		2	1		2	1		2	1		2	1
16	232	0		2	1		0	1	Minimal pa	1	1		0	1		2	1		1	0		2	0	Minimal pa	0	1		1	1		1	0
17	239	0		2	1		2	0	Minimal pa	2	1		2	1		2	1		2	1		2	1		2	1		2	1		2	1
18	246	0		2	1		2	0		2	1		2	1		2	1		2	0		2	1		2	1		2	1		2	1
19	247	0		2	1		2	1	No respon	2	1		2	0		2	0	Patient wi	2	0		2	0	Patient wi	2	0	Patient wi	2	0	Patient wi	2	0
20	248	0		2	1		2	0	Minimal pa	2	1		2	0		2	0		0	0		2	1	Minimal pa	1	1		2	1		2	0
21	263	0		2	1		2	1	Minimal pa	2	1		2	1		2	1		2	0		2	1		2	1		2	1		2	1
22	265	0		2	1		2	0		2	1		2	1		2	1		2	0		2	1	Minimal pa	1	1		2	1		2	0
23	267	0		2	1		2	0		2	1		2	0		0	1		2	0		2	1		2	1		2	1		2	0
24	275	0		2	1		2	0		2	1		2	0		2	1		2	0		2	1		2	1		2	1		2	1
25	281	0		2	1		2	1	Minimal pa	2	1		2	1		2	1		2	0		2	1		2	1		1	1		1	0
26	282	0		2	1		2	1		1	1		2	0		2	1		2	0		2	0	No respon	2	1	No respon	2	1	No respon	2	0
27	283	0		2	1		2	1	No respon	1	0		2	0		2	0		0	0		2	0		2	1		1	0		2	0
28	286	0		2	1		2	0		2	1		2	0		2	1		2	1		2	1		2	1		2	1		2	1
29	325	0		2	1		2	0	Minimal pa	2	1		2	0		2	1		1	0		2	0	Minimal pa	2	1		2	1		2	0
30	336	0		2	1		2	0	Minimal pa	2	1		2	1		2	0	Patient wi	2	0		2	1		2	1		2	1		2	1
31	369	0		2	1		2	1	Minimal pa	2	0	No respon	2	0		2	1		2	1		2	0	No respon	2	1	No respon	2	1	No respon	2	0
32	372	0		2	1		2	0		2	1		2	1		2	1		2	0		2	1		2	1		2	1		2	1
33	383	0		2	1		2	1	Minimal pa	2	1		2	0		2	1		2	0		2	1		2	1		2	1		2	1
34	386	0		2	1		2	0		2	1		2	0		2	1		2	0		2	1		2	1		2	1		2	1
35	397	0		2	1		2	0		2	1		2	0		2	1		2	1		2	1		2	1		2	1		2	0
36	405	0		2	1		2	1	Minimal pa	2	1		2	0		2	1		2	0		2	0	Minimal pa	1	1		1	1		1	0
37	406	0		2	1		1	0	Minimal pa	2	1		2	0		2	1		2	0		2	1	Minimal pa	1	1		2	1		2	0
38	407	0		2	1		2	0		2	1		2	0		2	1		2	1		2	1		2	1		2	1		2	1
39	408	0		2	1		2	1		2	1		2	0		2	1		2	0		2	1		1	1		2	1		2	1
40	414	0		2	1		2	0		2	1		2	1		2	1		2	0		2	1	Minimal pa	2	1		2	1		2	0
41	415	0		2	1		2	1	Minimal pa	2	0	No respon	2	1		2	1		2	1		2	0		2	1		2	1		2	0
42	420	0		2	1		2	0		2	1		2	0		2	1		2	1		2	1		2	1		2	1		2	0
43	430	0		2	1		2	0		2	1		2	0		2	1		2	0		2	1		1	1		2	1		2	0
44	436	0		2	1		2	0		2	1		2	0		2	1		2	0		2	1		2	1		2	1		2	0
45	439	0		2	1		2	0		2	1		2	0		2	1		2	0		2	1		2	1		2	1		2	1
46	468	0		2	1		2	0		2	0	Patient wi	2	0		2	0	Patient wi	2	0		2	0	Patient wi	2	0	Patient wi	2	0	Patient wi	2	0
47	475	0		2	1		2	1	Minimal pa	2	1		2	0		2	1		2	0		2	1		2	1		2	1		2	0
48	478	0		2	1		2	0		2	1		2	1		2	1		2	1		2	1		2	1		2	1		2	1
49	479	0		2	1		2	0		2	1		2	0		2	1		2	1		2	1		2	1		2	1		2	1
50	489	0		2	1		2	0		2	1		2	1		2	1		2	1		2	1		2	1		2	1		2	1
51	504	0		2	1		2	0		2	1		2	1		2	1	Patient wi	2	1		2	0		1	1		1	1		2	0
52	506	0		2	1		2	1	No respon	2	0	No respon	2	1		2	1		2	0		2	0	No respon	2	1		2	1		2	0
53	509	0		2	1		2	0		2	1		2	1		2	1		2	1		2	1		2	1		2	1		2	1
54	517	0		2	1		2	0		2	1		2	1		2	1		2	0		2	1		2	1		2	1		2	1
55	527	0		2	1		2	0		2	1		2	1		2	1		2	0		2	1		2	2		2	1		2	1
56	530	0		2	1		2	1		2	1		2	0		2	1		1	0		2	1		2	1		2	1		2	0
57	532	0		2	1		2	1		2	0		2	0		2	1		1	1		2	0	Minimal pa	2	1		2	1		2	0
58	535	0		2	1		2	1		2	0	No respon	2	0		2	0		0	0		2	0	No respon	2	1		2	1		2	0
59	542	0		2	1		2	1		2	1		1	1		2	1		1	0		2	0	Minimal pa	2	1		2	1		2	0
60	544	0		2	1		2	0		2	1		2	0		2	1		1	1		2	1		2	1		2	1		2	1
61	545	0		2	1		2	1		2	0	No respon	2	1		2	0		0	0		2	0	No respon	2	1		1	0	No respon	2	0
62	585	1		2	1		2	1		2	0		2	1		2	1		2	0		2	0	No respon	2	1		1	1		1	0
63	618	0		2	1		2	0		2	1		2	0		2	1		2	1		2	1		2	1		2	1		2	1
64	640	0		2	1		2	0		2	1		2	1		2	1		2	1		2	1		2	1		2	1		2	1
65	641	0		2	1		2	0		2	0	No respon	2	1		2	1		2	0		2	0	No respon	2	1		2	1	No respon	2	0
66	645	0		2	1		2	0		2			2	1		2	1		2	0		2	0	Patient wi	2	1	No respon	2	1	No respon	2	0
67	651	0		2	1		2	0		2	1		2	1		2	1		2	0		2	1		2	1		2	1		2	1
68	652	0		2	1		1	0		2	1		2	0		2	1		2	1		2	1		2	1		2	1		2	1
69	656	1		2	1		2	1	No respon	2	0		2	1		2	1		2	0	No respon	1	0	No respon	2	1		2	1		2	0

Guided discovery_ CBA model<br>Reason did not happen	Guided discovery_ CBA model<br>(0,1,2)	Optimises engagement<br>Did/dd not happen<br>0=no<br>1=yes	Optimises engagement<br>Reason did not happen	Optimises engagement<br>(0,1,2)	Number of messages sent per protocol	Number of messages patient to facilitator	Number of messages facilitator to patient
	118	67		128	64		
	85.5	97.1		92.8	92.8		
						5.2	10.1
Minimal pa	2	1		2	1	2	11
	1	1		2	1	15	13
Minimal pa	2	1		2	1	1	8
	2	1		2	1	7	14
	2	1		2	1	7	12
	1	1		2	1	9	14
	2	1		2	1	5	9
	1	1		2	1	6	8
	0	1		1	1	3	7
Minimal pa	2	1		2	1	3	9
	1	1		2	1	6	18
Minimal pa	2	1		2	1	2	8
	1	1		1	1	2	10
	2	1		2	1	6	10
	2	1		2	1	5	7
	2	1		1	1	0	6
	2	1		2	1	5	11
	2	1		2	1	7	11
Patient wit	2	1		2	1	0	3
Minimal pa	2	1		2	0	1	5
	2	1		2	1	1	10
Minimal pa	2	1		2	0	6	9
	0	1		2	0	4	5
	2	1		2	1	6	10
	0	1		1	1	4	9
No respon	2	1	No respon	2	0	0	6
	2	0		0	0	0	2
	2	1		2	1	14	11
Minimal pa	2	1		2	1	1	8
	1	1		2	1	6	11
No respon	2	1	No respon	2	1	1	10
	2	1		2	1	14	16
	2	1		2	1	3	13
	2	1		2	1	10	13
Minimal pa	2	1		2	1	8	13
	1	1		2	1	5	9
Minimal pa	2	1		2	1	4	12
	2	1		2	1	11	15
	2	1		2	1	2	11
Minimal pa	2	1		2	1	5	12
	2	1		2	1	1	9
	0	1		2	1	5	13
Minimal pa	1	1		1	1	3	12
Minimal pa	2	1		2	1	3	12
	2	1		2	1	12	15
Patient wit	2	0	Patient wit	2	1	2	2
	1	1		2	1	1	6
	2	1		2	1	15	17
	2	1		2	1	9	12
	2	1		2	1	15	16
	1	1		1	1	10	10
No respon	2	1		2	1	0	7
	2	1		2	1	5	13
	1	1		2	1	5	9
	2	1		2	1	10	11
Minimal pa	2	1		2	1	8	15
Minimal pa	2	1		2	1	2	9
No respon	2	1		2	1	2	10
Minimal pa	2	1		1	1	1	11
	2	1		2	1	12	11
No respon	2	1		2	1	0	6
Minimal pa	2	1		1	1	0	7
	1	1		2	1	18	12
	2	1		2	1	6	14
No respon	2	1		2	1	0	9
Patient wit	2	1	Patient wit	2	1	1	1
	2	1		2	1	11	13
	2	1		2	1	6	7
No respon	2	1		2	1	0	8
